# Evidence That Aquaporin 11 (AQP11) in the Spiny Dogfish (*Squalus acanthias*) May Represent a Pseudogene

**DOI:** 10.3390/ijms25042028

**Published:** 2024-02-07

**Authors:** Christopher P. Cutler, Meghan E. Canicatti, Esosa Omoregie

**Affiliations:** Biology Department, Georgia Southern University, P.O. Box 8042, Statesboro, GA 30460, USA

**Keywords:** aquaporin, spiny dogfish, pseudogene, degenerate PCR, colony PCR, 5′ RACE PCR, 3′ RACE PCR, amino acid alignment, nucleotide sequence alignment, DNA cloning

## Abstract

Various attempts to amplify an AQP11 cDNA from tissues of the spiny dogfish (*Squalus acanthias*) were made. Two pairs of deoxy-inosine-containing degenerate primers were designed based on conserved amino acid sequences from an AQP11 alignment. These primers yielded some faint bands from gill cDNA that were sequenced. Blast searches with the sequences showed they were not AQP11. An elasmobranch AQP11 nucleotide sequence alignment was produced to identify conserved regions to make further degenerate primers. One primer pair produced a short 148 bp fragment showing particularly strong amplification in gill and intestine. It was sequenced and represented a piece of the AQP11 gene. However, as the fragment may have resulted from contaminating genomic DNA (in total RNA used to make cDNA), 5′ and 3′ RACE were performed to amplify the two ends of the putative cDNA. Furthermore, 5′ and 3′ RACE amplifications depend on the presence of a 5′ cap nucleotide and a poly A tail, respectively on the putative AQP11 mRNA. Hence, successful amplification was only possible from cDNA and not genomic DNA. Nested RACE amplifications were performed using gill and intestinal RACE cDNA, but none of the DNA fragments sequenced were AQP11. Consequently, the spiny dogfish AQP11 gene may represent a pseudogene.

## 1. Introduction

Aquaporins (AQPs) are a ubiquitous family of membrane proteins found across the spectrum of life from bacteria to humans [1]. In mammals, there are thirteen aquaporins numbered AQP0-12 [2,3,4,5], whereas in other animals, there are up to seventeen AQP genes [1]. Mammalian aquaporins fall into three main groups, orthodox or classical aquaporins (AQP0, AQP1, AQP2, AQP4, AQP5, AQP6, and AQP8), aquaglyceroporins (AQP3, AQP7, AQP9, and AQP10), and unorthodox or superaquaporins (AQP11 and AQP12) [6,7]. AQP11 and AQP12 were originally identified and described as AQPX1 and AQPX2, respectively [8]. AQP11 in mammals is widely expressed in a number of tissues including brain, heart, liver, spleen, GI tract, kidney, testes, ovary, muscle, and leukocytes [3]. Mammalian AQP11 exhibits water permeability in expression studies [3], although it is additionally thought to represent a glyceroporin and a peroxiporin transporting hydrogen peroxide, and it localizes to the endoplasmic reticulum membrane internally within cells [9].

In teleost fish, there are duplicate copies of the AQP11 gene (AQP11a and AQP11b) [10]. In zebrafish (*Danio rario*), AQP11b is expressed in GI tract, liver, and ovary [10]. In American eel (*Anguilla rostrata*), AQP11a is expressed at the highest levels in liver, GI tract, and kidney, whereas AQP11b is found in the brain, gill, GI tract, and kidney with lower levels in the eye, heart, swim bladder, spleen, and liver [11].

Elasmobranchs, such as the spiny dogfish (*Squalus acanthias*), have a subset of the seventeen AQPs animals have, but with two individual gene duplications. Their AQP genomic complement includes AQP0, AQP1, AQP3C1, AQP3C2, AQP4, AQP8, AQP9, AQP10C1, AQP10C2, AQP11, AQP12, AQP14, and AQP15 [1,12,13,14,15,16,17]. All of these genes have now been cloned and sequenced from spiny dogfish cDNA except for AQP11 [18].

Until very recently, there has been very little sequence data available in gene banks regarding AQPs from elasmobranch species. Sequences for AQP11 from a number of elasmobranch species began to be submitted to gene banks in 2021 and those sequences helped facilitate this study. As no AQP11 sequence was available for the spiny dogfish, the initial purpose of the study was to obtain a copy of the coding region of the AQP11 mRNA transcript using complementary DNA (cDNA) derived from various tissue total RNA samples, and using degenerate PCR techniques successfully deployed many times [14,15,17,19,20,21,22,23,24,25]. With AQP11 sequences from closely related shark species available, it was initially thought that it would be a relatively easy and straight forward task to amplify a piece of the spiny dogfish AQP11 cDNA, but that proved to be far from the reality of the situation.

## 2. Results

### 2.1. Amino Acid-Based Degenerate PCR

**Figure 1:**
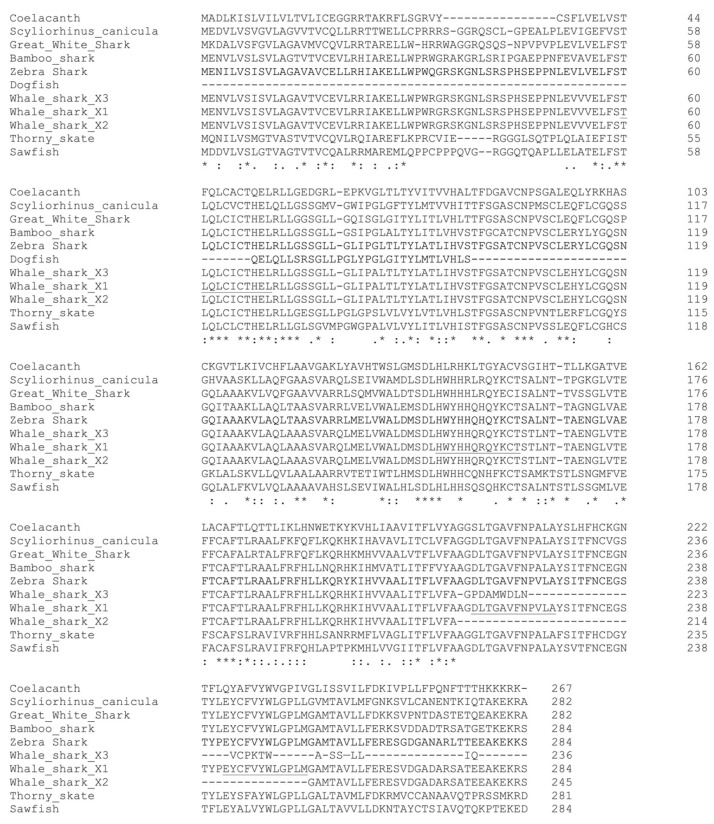
Clustal Omega alignment of the amino acid sequences of elasmobranch and coelacanth AQP11 amino acid sequences. The alignment includes a translation of the partial spiny dogfish AQP11 sequence resulting from this study. Underlining in the whale shark X1 sequence represents locations of conserved amino acid sequences used to design amino acid-based degenerate PCR primers (see also Table 1). * indicate locations in the alignment with the same amino acid in all species and : or . indicate positions in the alignment with chemically similar substitutions in some species.

Initial studies to amplify a piece of the spiny dogfish AQP11 transcript from cDNA utilized an established degenerate PCR technique. Amino acid sequences from various elasmobranch and coelacanth AQP11 genes were aligned, and conserved stretches of sequences were identified to allow for the design of inosine-containing primers (Figure 1; see also Section 4).

**Figure 2:**
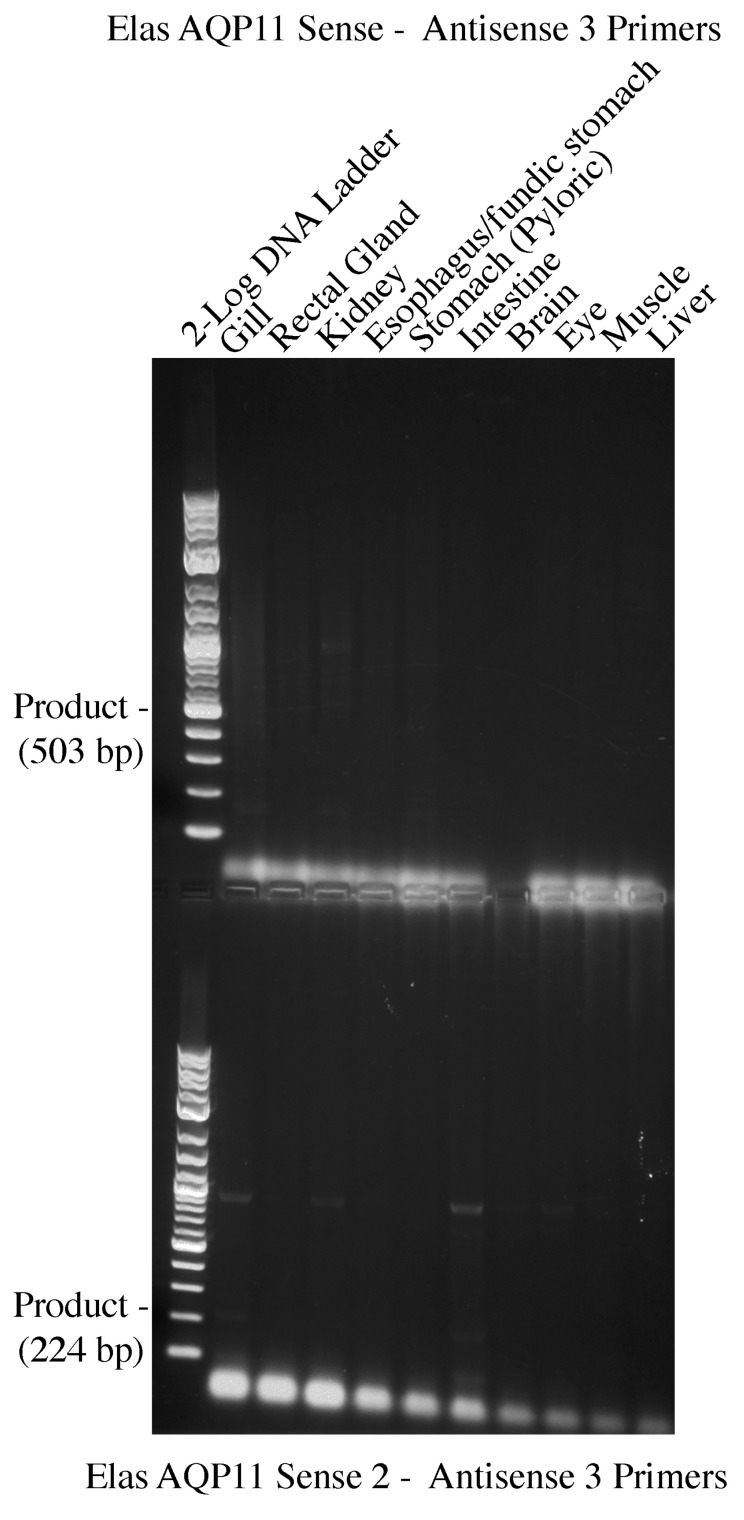
An example two row 1.5% agarose electrophoresis gel of amino acid-based AQP11 degenerate PCR amplifications performed with two different combinations of primers; Elas AQP11 sense—Elas AQP11 Antisense 3, (**top**); Elas AQP11 Sense 2—Elas AQP11 Antisense 3, (**bottom**). PCR reactions were carried out on a range of ten tissue cDNAs (as labelled above each track) from the spiny dogfish. The 2-log DNA marker ladder (NEB) was used for band size analysis. The expected sizes of DNA products generated with each primer pair are shown at the left side of each part of the gel.

Initially, one pair of primers were made (Elas AQP11 Sense and Elas AQP11 Anti 2). These primers should have resulted in an approximate 578 bp fragment, which is generated from various spiny dogfish tissue cDNAs in degenerate PCR. An initial PCR run using an annealing step of 55 °C resulted in no bands around the correct size in any tissue. A second run was performed using an annealing step of 50 °C. This resulted in two faint bands in the gill, but when cloned and sequenced, these transpired to be unrelated to AQP11. A further pair of degenerate primers were then made using amino acid sequences from other regions of the AQP11 gene (Elas AQP11 Sen 2 and Elas AQP11 Anti 3). These new primers were used in conjunction with the original primers, in addition to being used on their own (possible combinations include Sense-Anti 3, Sen 2-Anti 2, and Sen 2-Anti 3). An example gel resulting from some of the amplifications performed with these primers at 55 °C is seen in Figure 2. Although some bands were seen with the gill cDNA and cloned and sequenced (three bands), none of these proved to be related to AQP11. It should be noted, at this point, that both of the sense primer sites were located within the expected first exon of the gene, whereas the antisense primers were mostly or entirely within the expected second exon of the gene; therefore, it was only possible to form AQP11 amplification products of the expected size from the cDNA and not from any genomic DNA contamination present in the total RNA/cDNA.

### 2.2. Nucleotide-Based PCR

**Figure 3:**
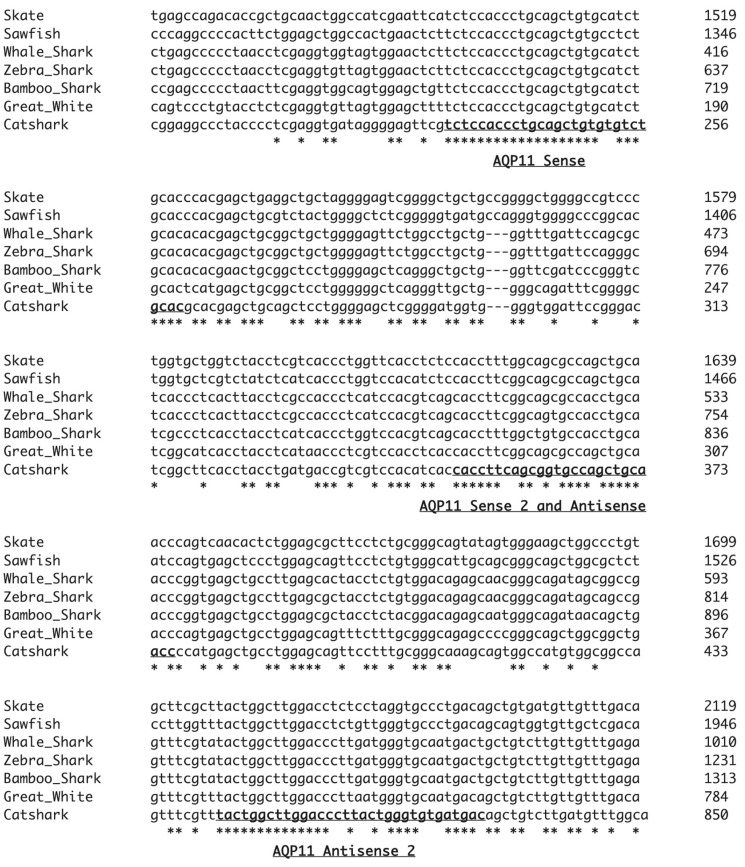
Partial Clustal Omega alignment of seven elasmobranch AQP11 cDNA sequences, showing only the relevant alignment blocks used to design nucleotide-based degenerate primers. Primer locations are indicated with underlined and bolded text in the catshark sequence. Names of the AQP11 primers produced from each region are indicated underneath alignment blocks. * indicate positions where the nucleotide sequence is identical in all species.

**Figure 4:**
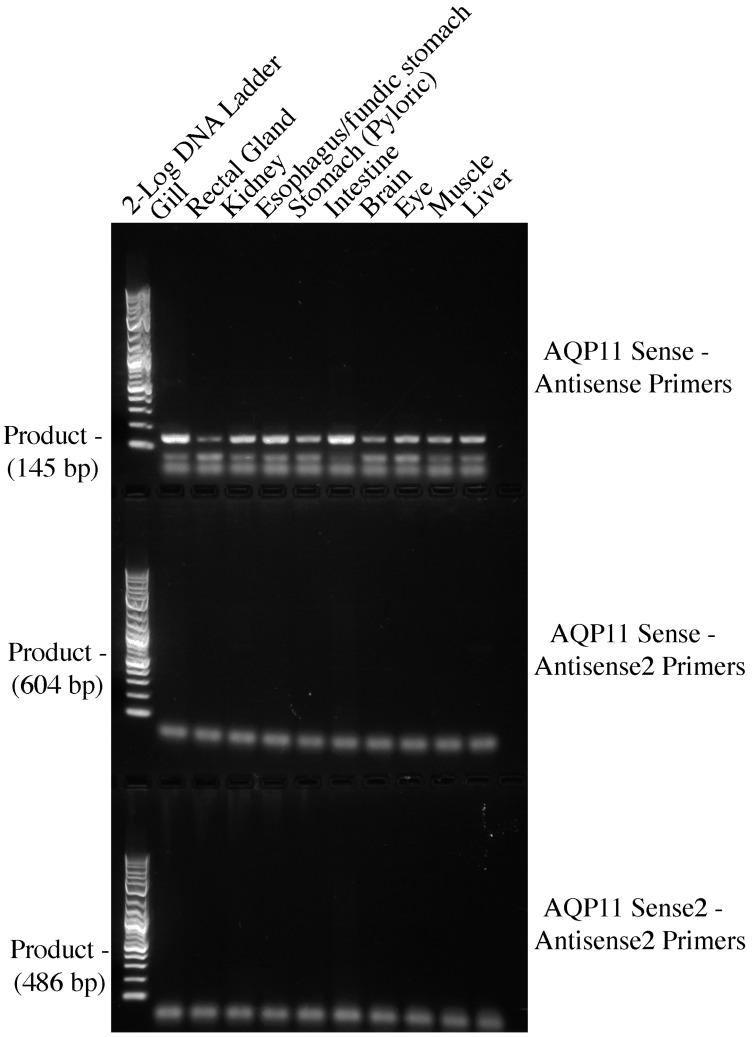
A three row 1.5% agarose electrophoresis gel showing three sets of nucleotide-based degenerate primer amplifications using ten cDNAs from a range of spiney dogfish tissues (labelled at the top of each track). The 2-log DNA marker ladder (NEB) was used with each set of amplifications for band size analysis. Expected DNA product sizes are indicated at the side of each set of amplifications.

Although this initial series of amino-acid-sequence-based degenerate PCRs suggested that there was no expression of AQP11 in any of the spiny dogfish tissue cDNA samples, given that the shark AQP11 sequences appeared to be fairly highly conserved, it was decided to attempt to obtain an AQP11 amplification product using nucleotide sequence-based degenerate PCR primers. The nucleotide sequences of various shark AQP11 genes were aligned and three conserved regions were identified (see Figure 3 and Table 2). With three sites available, both sense and antisense primers were designed for the middle site such that three sets of PCR amplifications could be performed (AQP11 sense-AQP11 antisense, AQP11 sense-AQP11 antisense 2, AQP11 sense 2-AQP11 antisense 2; see Figure 4). 

**Figure 5:**
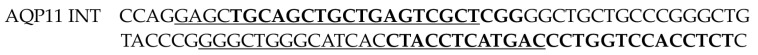
Nucleotide sequence of the partial spiny dogfish AQP11 148 bp fragment generated with the AQP11 sense and AQP11 antisense nucleotide-based degenerate PCR primers (see Table 2). The primer sequences themselves have been removed as they are unreliable. The 5′ RACE primers produced from this sequence are bolded, and the 3′ RACE primers are underlined.

With the set of amplifications performed using the AQP11 sense-AQP11 antisense primers, a band of approximately the expected size of 145 bp (actually 148 bp) was identified in every tissue cDNA. The highest level of amplification was seen in the gill and intestine; therefore, these bands were cloned and sequenced. The fragments were essentially copies of a piece of the spiny dogfish AQP11 gene (see Figure 5).

However, it was also evident that both of the primers were used to make the successful fragment reside within the expected exon 1 of the AQP11 gene. Hence, the fragment could have amplified from either the cDNA or any contaminating genomic DNA within the cDNA samples.

### 2.3. Race PCR

In an attempt to determine whether the AQP11 fragment obtained was from a cDNA or genomic DNA origin, primers were designed from the obtained AQP11 sequence for RACE PCR in order to amplify the two ends of the cDNA sequence. The RACE PCR relies for its success on the presence of a poly A tail on the 3′ end of the mRNA and a 5′ cap on the other end of the mRNA. Without the presence of these nucleotide structures, the cDNA would fail to be produced and the RACE PCR would then subsequently fail to amplify AQP11 fragments. Of course, the poly A tail and 5′ cap nucleotide are features of the mRNA added enzymatically after transcription and are not present within the genomic copy of genes. Hence, RACE PCR differentiates between sequences amplified from cDNA and genomic DNA and will not produce amplification products from the genomic copies of genes.

**Figure 6:**
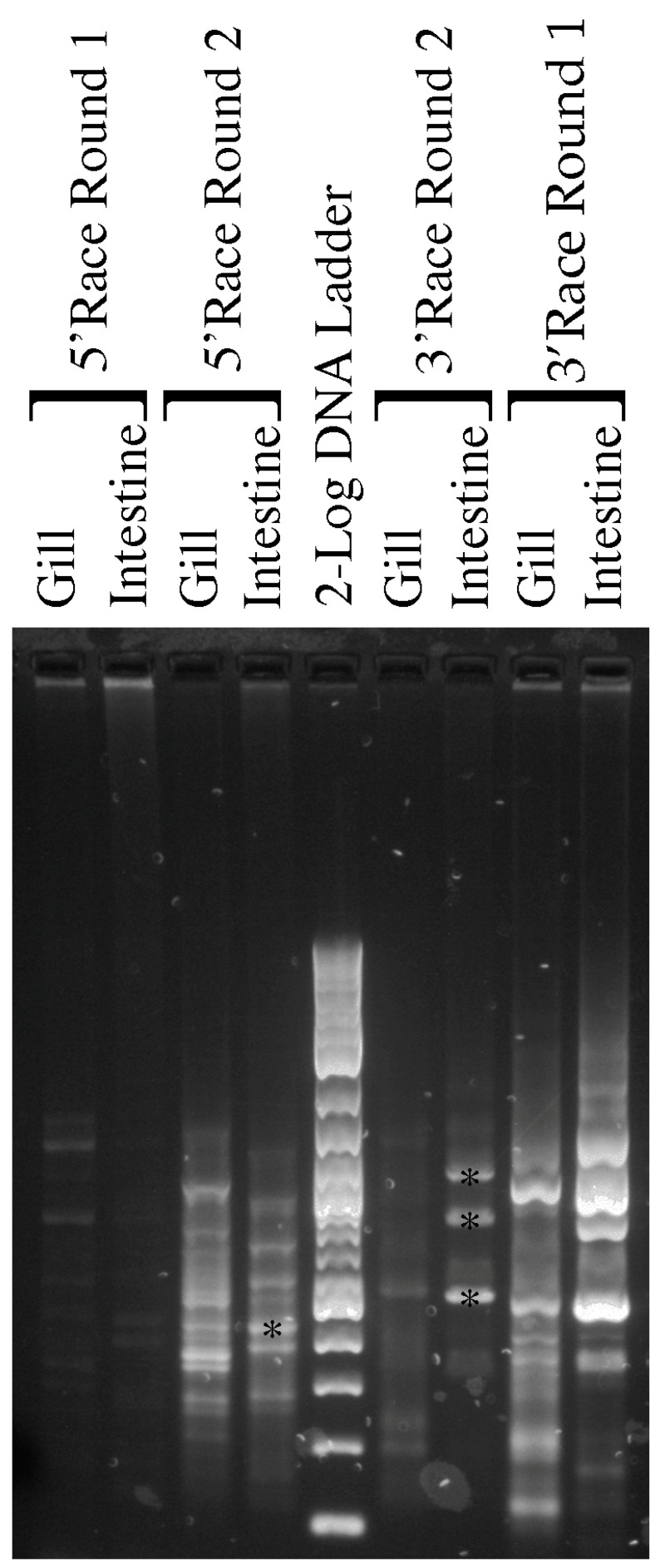
A 1.5% agarose gel of Nested 5’ and 3’ spiny dogfish AQP11 RACE products produced from gill and intestinal 3’ and 5’ RACE cDNA. The first round of nested PCR reactions is listed as Round 1. These Round 1 reactions were then used as the DNA templates for Round 2 nested PCR reactions.2-log DNA marker ladder (NEB) is shown as a size reference. Bands in intestinal cDNA Round 2 tracks marked with a * were excised, purified, cloned and sequenced.

Nested RACE PCR was performed (see Figure 6 and Table 3) and while some strong bands occurred particularly in the first round of 3′ RACE amplification, the band in the second round was diminished rather than increased as would be expected. The three main intestinal 3′ RACE bands were cloned and sequenced. In the resulting sequences, two sizes of clones were present. Both bands had utilized the AQP11 gene specific primer sequences (3R1) from the first round of RACE amplification to amplify (i.e., they were first not second-round amplification products) and blast searches with the sequences showed that both bands were angiotensin-converting enzyme (ACE) splice variants, which are most closely related to those from the bamboo shark and zebra shark, respectively.

For 5′ RACE, the bands did increase in intensity in the second round of amplification. To amplify to the end of the transcript, there was a minimum expected size of around 260 bp. Also, as the primers for the second round of nested RACE PCR are internal to the initial round of AQP11 fragments, the size of AQP11 should decrease by a known amount (73 bp) in the second round of nested RACE PCR. Therefore, one band from the intestinal cDNA was chosen for cloning and sequencing as it was the strongest amplifying band on the gel and there was a somewhat larger band in the initial round of amplifications. When the cloning experiment was analyzed, two sizes of clones were present (417 bp and 368 bp), which were both sequenced. Additionally, blast searches with the sequences were most similar to genomic DNA on catshark chromosome 21 and great white shark dual specificity phosphatase 5 (dusp5), respectively.

In summary, none of the sequenced 5’ or 3’ RACE products represented AQP11 sequences.

## 3. Discussion

It was surprising that the amino acid-based degenerate PCR failed to generate an AQP11 PCR product since the AQP11 gene in elasmobranchs appears to be fairly highly conserved. Therefore, AQP11 should have represented a relatively easy target for this approach, as the AQP8 gene [17] had been previously, even though the AQP8 study did not have any prior elasmobranch sequences to work with. Additionally, AQP11 expression was expected to be present in most of the tissue cDNAs utilized in this study, such as brain, liver, gill, GI tract, and kidney, based on the AQP11 expression profile in mammals (such as in humans [3]) or in teleost fish (such as in the American eel [11]). Therefore, the lack of amplification in the amino acid-based degenerate PCRs was unexpected but would be comprehensible if AQP11 is a pseudogene because the primers used span across the expected exons of the gene. As a result, potential products generated from genomic copies of the gene would be much larger (due to the presence of an intron). A second line of circumstantial evidence that is in line with this study is the fact that a transcriptomics study carried out on spiny dogfish cDNAs from brain, kidney, liver, and ovary failed to identify an AQP11 transcript [26]. On the other hand, the transcriptomics study seems to have not been ultimately exhaustive in identifying every transcript present in these tissues as AQP8, which was subsequently shown to be expressed in spiny dogfish brain [17], was not identified either. The amplification of a small AQP11 fragment by one of the nucleotide-based degenerate PCR primers might have suggested that AQP11 expression was present in spiny dogfish tissue cDNAs, but it was known in advance that the total RNA samples used to make cDNAs were contaminated with a small amount of genomic DNA and the fragment generated (see Figure 5) would be contained within the expected exon 1 of the genomic copy or the AQP11 gene and would amplify with the primers used. The AQP11 antisense 2 primer would be expected to be located in exon 2 of the AQP11 gene, and therefore this primer would only produce a product of the expected size from the cDNA version of AQP11. Once again, the failure of the AQP11 antisense 2 primer to produce PCR products of the expected size in any tissue is consistent with a lack of AQP11 expression in these tissues, which would be the case if AQP11 is a pseudogene. Finally, the RACE amplification using primers designed from the spiny dogfish AQP11 fragment generated (see Figure 5) again would only likely amplify from cDNA copies of AQP11 because the cDNA synthesis reactions that incorporate a Smarter DNA sequence (see methods) rely on the binding of an oligo dT portion of the cDNA synthesis primer to bind to the AQP11 mRNA poly A tail, and for 5′ RACE, the incorporation of the Smarter oligo sequence at the 5′ end relies on the presence of a 5′ cap nucleotide on the mRNA. Both poly A tail and 5′ cap nucleotide are only present on mRNAs and are not found within the genomic sequences of genes. This is the reason why it would be unlikely that 5′ and 3′ RACE AQP11 fragments would be generated by the RACE reactions unless the AQP11 gene was transcribed into the mRNA.

Ultimately, this study is not proof that expression of the AQP11 gene in the spiny dogfish does not exist. In mammals, the highest level of AQP11 expression is in the testes [3] (although in zebrafish no expression was found; [10]). Since this study did not have access to any gonadal total RNA/cDNA samples, this is impossible to rule out. Similarly, in any case, AQP11 expression might be limited to other organs that are not utilized in this study, such as pancreas or in tissues, which are not part of major organs such as cartilage or in the integument. It is, however, interesting to note that in a recent article concerning the super aquaporin subfamily [9], the authors noted that due to the lack of significant AQP11 phenotypes in knock-out animals, “that AQP11 is dispensable similar to AQP10 which has turned to a pseudogene in some animals”. The reference to AQP10 refers to its lack of expression in cows and their relatives [27].

## 4. Methods

### 4.1. cDNAs

The total RNA/cDNAs used in this study were the same as those in [28]. Complementary DNAs (cDNAs) were made by a modification of the manufacturer instructions. One μg of previously extracted total RNA samples from the tissues of an adult spiny dogfish (when the animal was originally euthanized in 2004, the work had received IACUC approval from the Georgia Southern University and the Mount Desert Island Bio. Lab., Bar Harbor, ME, USA) were incubated with 1 μL of 100 μM Oligo d (T)_26_ primer, 1 μL of 10 mM dNTPs (Lamda Biotech, Saint Louis, MO, USA), 1 μL of SUPERas.in RNase inhibitor (Thermofisher, Waltham, MA, USA), and dH_2_O to a total of 6 μL. The samples were then mixed and incubated at 65 °C for 5 min, and then were placed on ice for 1 min. Thereafter, 2 μL of 5x RT buffer and 1 μL 100 mM dithiothreitol (DTT) were added and the samples were mixed by pipetting. Finally, 1 μL (200 units) of Superscript IV reverse transcriptase (Thermofisher) was added and the samples were mixed and incubated at 55 °C for 30 min. The samples were then incubated at 80 °C to inactivate the reverse transcriptase, and then they were placed on ice and diluted to 200 μL with dH_2_O.

### 4.2. Amino Acid-Based Degenerate PCR

AQP11 degenerate PCR primers were designed based on reverse-translated conserved amino acid sequences, as shown in Figure 1. Gene alignments were performed using Clustal Omega (at www.ebi.ac.uk, accessed on 25 March 2023) and the sequence of the spiny dogfish DNA fragment was translated using an online Translate tool (web.expasy.org/translate/, accessed on 25 March 2023). Deoxy-inosine was used at positions of uncertainty in the sequences (often the third base of codons; see Table 1). Primers were manufactured by Eurofins Genomics (Louisville, KY, USA). Degenerate PCR was performed using an Eppendorf (Framingham, MA, USA) Mastercycler pro S thermocycler. For each primer pair, reactions were carried out initially with a cycle annealing temperature of 55 °C, and then subsequently at 50 °C. Cycle extension times at 72 °C were approximately 1 min/kbp of DNA fragment. Denaturation during each cycle was carried out at 94 °C for 30 s. PCR reactions contained 1 μL of each 100 μM degenerate primer and 1 μL of cDNA as separate drops in a 200 μL reaction tube. A master mix containing 2 μL 10X Taq polymerase buffer (NEB, Ipswich, MA, USA), 0.4 μL 10 mM dNTPs, 0.25 μL Taq DNA polymerase (NEB), and dH_2_O to 17 μL was created and added to PCR reaction tubes immediately before they were added to the thermocycler, which was paused at 92 °C. Once all the tubes were added to the thermocycler, its cycling program was initiated for 40 cycles.

### 4.3. Agarose Gel Electrophoresis

A 135 mL (1.5% *w*/*v* agarose gel was produced. Then, 2.025 g agarose gel (Lamda Biotech) was weighed into a conical flask and 132 mL dH_2_O was added to 2.7 mL 50X TAE buffer. The mouth of the flask was covered with a cling film poked with holes to release steam. The flask was heated on full power for 45 s, and the contents were swirled and microwaved for a further 1 min on 30% power. The flask was placed in a water bath to cool at >45 °C. Thereafter, 13.5 μL of 10,000X gel red (Biotium, Fremont, CA, USA) electrophoresis dye was added and mixed by swirling. The gel was poured into a taped (masking taped ends) gel tray. Gel well combs were added to the gel and it was allowed to set. The gel was placed in a fridge for >20 min after setting before removal of the comb(s). PCR samples were added to the wells. A 2-log ladder DNA marker sample (NEB) was prepared with 1X TAE buffer and bromophenol blue loading dye. Samples were run initially for up to 2 min at 500 mAmps, then subsequently at a constant 265 mAmps. Finally, the gels were visualized and photographed using a Syngene (Cambridge, UK) G box gel documentation system.

### 4.4. Cloning and Sequencing

DNA bands were excised from agarose gels using a No. 11 scalpel blade and DNA was purified using a Monarch DNA purification kit (NEB). Purified DNA was cloned into a pCR4 TOPO plasmid vector hosted in *E. coli* using a TOPO TA Cloning Kit for sequencing (Thermofisher). Bacterial colonies from LB agar plates were grown in 1 mL Terrific Broth, and then processed. Thereafter, 50 μL of bacterial cultures was centrifuged and the culture medium was removed. The bacteria were resuspended in 0.5 mL dH_2_O and 0.5 μL of each suspension was used for colony PCR. Colony PCR reactions were performed with 1 μL each of 4 μM M13 forward and reverse plasmid primers, which also contained 2 μL 10X Taq polymerase buffer, 0.4 μL 10 mM dNTPs, and 0.1 μL (0.5 units) Taq DNA polymerase. Thereafter, 4 μL of each colony PCR sample was run on agarose gels and the best three were selected for sequencing. The remains of the colony PCR samples selected were purified using a Quickstep 2 PCR purification kit (Edge Biosystems, San Jose, CA, USA). Then, 1 μL of the purified DNA was run on another agarose gel with Logic DNA marker ladder (Lamda Biotech) and the amount of DNA in samples was quantified using the Syngene G box gel documentation system. Finally, 20 ng/kb of each DNA sample was placed in a 200 μL PCR tube, 1 μL of 4 μM T3 plasmid primer was added, and the volume was made up to 8 μL with dH_2_O. Samples were then sent for sequencing (Eton Biosciences, Research Triangle Park, NC, USA).

### 4.5. Nucleotide-Based Degenerate PCR

Primers were designed (manufactured by Eton Biosciences, Research Triangle Park, NC, USA) using conserved regions of an alignment of the AQP11 nucleotide sequences from various elasmobranch species (see Figure 3 and Table 2). Primers were made sufficiently long to allow for the use of 2-step PCR using Phusion DNA polymerase (NEB). The cycle program involved initial denaturation for 30 s at 98 °C followed by 35 cycles of 72 °C for 30 s/kb (+8 s), then 98 °C for 10 s per cycle. There was then a final extension step of 2 min at 72 °C.

### 4.6. RACE PCR

Pre-existing spiny dogfish tissue 5′ and 3′ RACE cDNAs were used for the study, which were manufactured using a SMARTer RACE cDNA synthesis kit (Takara Bio, San Jose, CA, USA) and according to the manufacturer’s instructions, except for the kit’s reverse transcriptase steps, which were replaced by those needed for Superscript IV reverse transcriptase and SUPERas.in RNase inhibitor. Some of the cDNAs generated had been previously used successfully for other genes (for example, see [15,17]). The cDNAs for 5′ RACE and 3′ RACE are made separately and a Smarter oligo sequence is incorporated at the 5′ cap in 5′ RACE cDNA and as the rear part of the oligo dT cDNA synthesis primer in 3′ RACE cDNA. PCR is conducted using a Universal Smarter RACE primer mix from the kit and a gene specific primer (5R1 for 5′ RACE or 3R1 for 3′ RACE; see Table 3). According to the manufacturer’s instructions, initial (Round 1) PCR amplifications were conducted using touchdown PCR with an initial annealing temperature for 5 cycles at 72 °C, then at 70 °C for 5 further cycles, and then at 68 °C for a further 25 cycles. The polymerase kits were replaced by Phusion DNA polymerase (NEB) and the extension time at 72 °C was 2 min per cycle. The PCR reactions generated were then used to seed a second set of nested PCR reactions (Round 2) using the kit’s nested primer (Universal primer short) and a second nested gene specific primer (5R2 for 5′ RACE or 3R2 for 3′ RACE; see Table 3). These reactions were performed for 35 cycles using 98 °C denaturation for 10 s and 2 min at 72 °C (a 2-step PCR approach), followed by a final extension step of 5 min at 72 °C.

### 4.7. Sequence Accession Numbers

Coelacanth AQP11, XM_006006304; Catshark AQP11, XM_038818680; Great white shark AQP11, XM_041199754; Bamboo shark AQP11, XM_043692428; Zebra shark AQP11, XM_048532354; Dogfish AQP11, PP214910; Whale shark AQP11 X1, XM_020529003; Whale shark AQP11 X2, XM_048597661; Whale shark AQP11 X3, XM_020529003; Thorny skate AQP11, XM_033023374; Sawfish AQP11, XM_052026755 Great white shark dusp5, XM_41209980; Catshark chromosome 21, LR744050.1; Bamboo shark ACE, XM_043674648.1; Zebra shark ACE, XM_059638649.1.

## Figures and Tables

**Table 1:** Amino acid-based degenerate PCR primers.

Elas AQP11 Sense	AC[I]YT[I]CARYT[I]TGYATHTGYAC[I]CAYGARYT
Amino Acid sequence used	TLQLCICTHEL
Elas AQP11 Anti 2	AR[I]GG[I]CC[I]ARCCARTA[I]ACRAARCARTAYTC
Amino Acid sequence used	EYCFVYWLGPL
Elas AQP11 Sen 2	CAYTGGTAYCAYCAYCARCR[I]CARTAYAARTG
Amino Acid sequence used	HWYHHQR/HQYKC
Elas AQP11 Anti 3	AR[I]RC[I]GGRTTRAA[I]AC[I]GC[I]CC[I]GT[I]ARRTC
Amino Acid sequence used	DLTGAVFNPV/AL
Expected product sizes:	Sense-Anti 2 = 578 bp
	Sense-Anti 3 = 503 bp
	Sen 2-Anti 2 = 299 bp
	Sen 2-Anti 3 = 224 bp

[I] = Deoxyinosine. Other IUPAC codes used indicate wobbles, e.g., R = A/G.

**Table 2:** Nucleotide-based degenerate PCR primers.

AQP11 Sense	TCTCCACCCTGCAGCTGTGYRTCTGCAC
AQP11 Sense 2	CACCTTYRGCDGYGCCASCTGCAAYCC
AQP11 Antisense	GGRTTGCAGSTGGCRCHGCYRAAGGTG
AQP11 Antisense 2	GTCATYRCACCYADYARRGGTCCAAGCCAGTA
Expected product sizes:	Sense-Antisense = 145 bp
	Sense–Antisense 2 = 604 bp
	Sense 2-Antisense 2 = 486 bp

IUPAC codes used indicate wobbles, e.g., R = A/G.

**Table 3:** The 5′ and 3′ RACE PCR primers.

5R1	AGAGGTGGAC CAGGGTCATG AGGTAG
5R2	CCGAGCGACT CAGCAGCTGC A
3R1	GAGCTGCAGC TGCTGAGTCG CT
3R2	GGGCTGGGCA TCACCTACCT CATGAC
Minimum expected product sizes:	
5′ RACE	=260 bp
3′ RACE	=632 bp

## Data Availability

The data is contained within this article. Figure images can be provided upon request.

## Abbreviations

AQP	Aquaporin
cDNA	Complementary DNA
RACE	Rapid Amplification of cDNA Ends
PCR	Polymerase Chain Reaction
Elas	Elasmobranch
ACE	Angiotensin Converting Enzyme
Dusp5	dual specificity phosphatase 5
RNase	Ribonuclease
DTT	dithiothreitol
dNTP	De-oxy nucleotide tri-phosphates
Taq	*Thermus aquatus*
IUPAC	International Union of Pure and Applied Chemistry
*E. coli*	*Escherichia coli*
NEB	New England Biolabs
